# 1954. Dynamic PET facilitated Modeling and Novel Antibiotic Regimens for Tuberculous Meningitis due to Multidrug-Resistant Strains

**DOI:** 10.1093/ofid/ofad500.108

**Published:** 2023-11-27

**Authors:** Xueyi Chen, Oscar J Nino-Meza, Mona O Sarhan, Bhavatharini Arun, Byeonghoon Jeon, Alvaro A Ordonez, Laurence S Carroll, Charles A Peloquin, Joel Freundlich, Vijay Ivaturi, Sanjay K Jain

**Affiliations:** Johns Hopkins University School of Medicine, Baltimore, MD; Johns Hopkins University School of Medicine, Baltimore, MD; Johns Hopkins University School of Medicine, Baltimore, MD; Pumas AI, Inc, Centreville, Virginia; Johns Hopkins University School of Medicine, Baltimore, MD; Johns Hopkins, Baltimore, Maryland; Johns Hopkins University School of Medicine, Baltimore, MD; University of Florida College of Pharmacy, Gainesville, Florida; Rutgers New Jersey Medical School, Newark, New Jersey; University of Maryland School of Pharmacy, Baltimore, Maryland; Johns Hopkins, Baltimore, Maryland

## Abstract

**Background:**

Tuberculous meningitis is the most serious form of tuberculosis (TB), affecting the young and immunocompromised. TB meningitis due to multidrug-resistant (MDR) strains is associated with high mortality (40-100%). Importantly, effective treatments for MDR TB meningitis are lacking, and the activity of the only approved MDR regimen for pulmonary TB (BPaL – bedaquiline, pretomanid, linezolid) is substantially inferior to the standard TB regimen in a mouse model of TB meningitis.

**Methods:**

Dynamic positron emission tomography (PET) was performed using radioanalogs of antibiotics (^18^F-pretomanid, ^18^F-sutezolid, ^18^F-linezolid and ^76^Br-bedaquiline) active against MDR strains to measure multicompartmental exposures (area under the curve, AUC). Each radioanalog is chemically identical to the parent antibiotic and the radioisotope is retained within the major metabolite. PET facilitated pharmacokinetic modeling predicted tissue exposures which were used to design optimized regimens (Fig.1).

**Figure 1.**
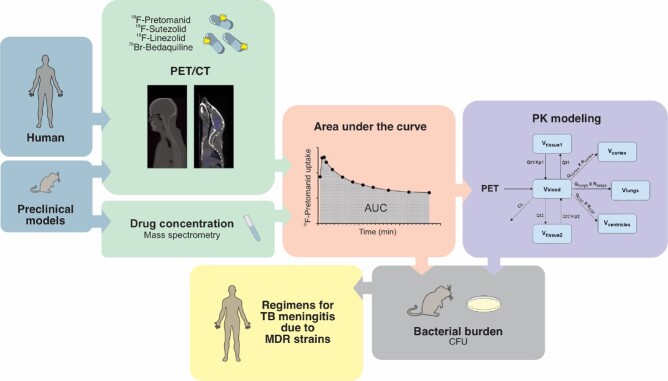
Study design.

Dynamic positron emission tomography (PET) was performed using radioanalogs of antibiotics (18F-pretomanid, 18F-sutezolid, 18F-linezolid and 76Br-bedaquiline) active against MDR strains to measure multicompartmental antibiotic exposures (area under the curve, AUC) in mouse models of TB. First-in-human 18F-pretomanid PET studies were performed in six subjects. PET signal was quantified by drawing volumes of interest (VOI) in brain, lung, and left ventricles (blood, converted to plasma) to measure antibiotic exposures [area under the concentration time curve (AUC)] represented as AUCtissue/plasma ratios. Pharmacokinetic analyses were performed to predict antibiotic exposures at human equipotent dosing in brain tissues, which were used to design optimized pretomanid-based regimens for TB meningitis. These optimized regimens were evaluated in the mouse model of TB meningitis. Approvals from the Johns Hopkins Biosafety, and Radiation Safety were also obtained for all studies.

**Results:**

PET demonstrated discordant antibiotic exposure in lung and brain compartments in mouse studies. While all antibiotics achieved high lung exposures (AUC_lung/plasma_ ∼1), only ^18^F-pretomanid achieved high brain exposures (Fig. 2). First-in-human ^18^F-pretomanid PET studies (n = 6 subjects) also demonstrated high exposures in both lung and brain compartments (Fig. 3). Pharmacokinetic modeling confirmed equivalence between mouse and human PET studies and identified the human pretomanid dose necessary to attain therapeutic brain exposures. Several pretomanid-based regimens demonstrated excellent bactericidal activity, equivalent or better than the standard TB regimen in the mouse model of TB meningitis, with addition of pyrazinamide significantly improving the activity of all regimens (Fig. 4).

**Figure 2.**
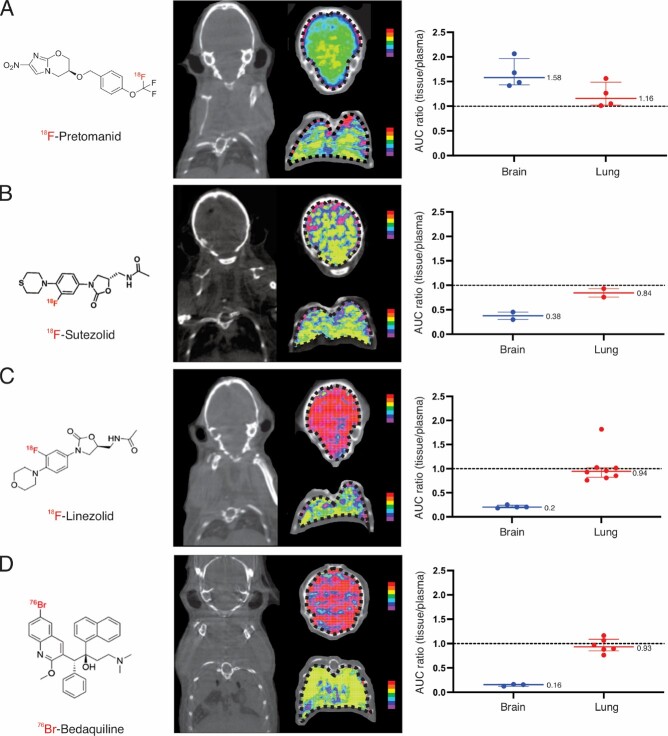
Antibiotic exposure in lung and brain compartments in mouse studies.

Radioanalog of four antibiotics active against multidrug-resistant (MDR) Mycobacterium tuberculosis strains were utilized in mouse studies. Each radioanalog is chemically identical to the respective parent antibiotic and the radioisotope is retained within the major metabolite enabling visualization of both the parent antibiotic and the metabolite by PET. Coronal PET AUC heat maps demonstrating spatially compartmentalized antibiotic exposures in lung and brain compartments are shown for (A) 18F-pretomanid, (B) 18F-sutezolid, 18F-linezolid and (D) 76Br-bedaquiline. 18F-Linezolid and 76Br-bedaquiline levels were derived from previously published studies (Mota et al. ACS Infect. Dis 2020 and Ordonez et al. ACS Infect. Dis. 2019). Antibiotic exposures are corrected for tissue density. Plasma data were obtained from blood (left ventricle) by correcting for hematocrit and RBC partitioning (except bedaquiline where RBC partitioning data is unavailable). PET studies are based on microdoses (ng- μg) administered intravenously. Data are represented as median ± interquartile range. Animal studies were approved by the Johns Hopkins Animal Care and Use Committee.

**Figure 3.**
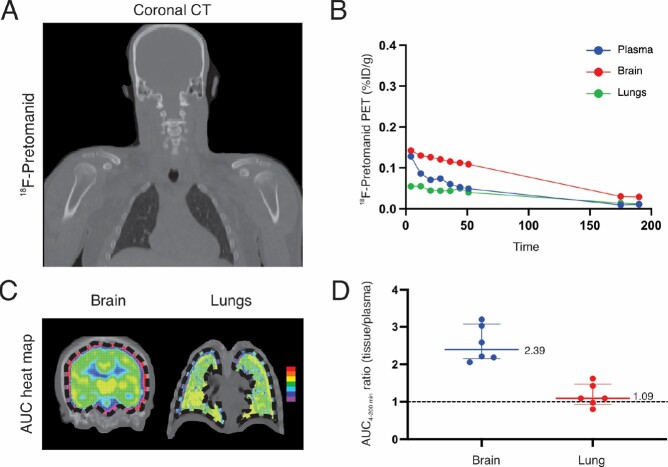
First-in-human 18F-pretomanid PET studies.

Six healthy subjects were prospectively recruited at the Johns Hopkins Hospitals and 18F-pretomanid PET was performed in accordance with U.S. Food and Drug Administration (FDA) guidelines for investigational drugs. (A-C) Coronal computed tomography (CT) (A) and time-activity curves (TAC) (B) and AUC heat maps demonstrating spatially compartmentalized antibiotic exposures in lung and brain compartments (C) from a representative human subject are shown. (D) AUC4-200 min (tissue/plasma) ratios from lung and brain compartments are shown for all six subjects as median ± interquartile range. Human studies were approved by the Johns Hopkins University Institutional Review Board Committee.

**Figure 4.**
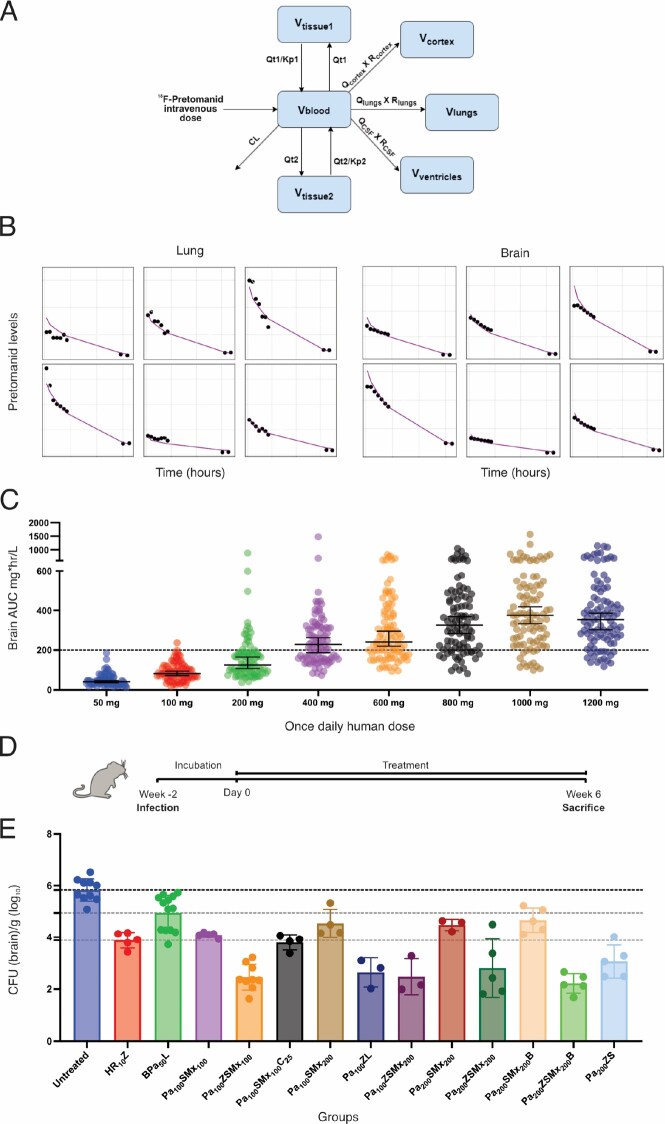
Optimized regimens for multidrug-resistant TB meningitis.

Pretomanid has concentration-time dependent activity and doses up to 1,200 mg (6x higher than used currently) are well tolerated in humans (Diacon et al. Antimicrob Agents Chemother. 2010), providing an opportunity to optimize the pretomanid dosing strategy. (A) Schematic representation of the pharmacokinetic model to predict tissue exposures. (B) Observed (black dots) and model-predicted (purple lines) pretomanid lung and brain tissue concentrations. (C) Human brain pretomanid exposures (AUC) predicted using Monte Carlo simulations for oral doses administered once daily. (D) Mice were infected intraventricularly via burr hole using a syringe and stereotaxic instrument (Ruiz-Bedoya et al. J Clin Invest. 2022). Oral antibiotic treatments (via oral gavage) at human equipotent dosing were initiated two weeks after infection (day 0) and administered for six weeks. After completion of antibiotic treatments, mice were sacrificed, tissue were homogenized and colony forming unit (CFU) were enumerated ex vivo. Mouse dosing was utilized to match the standard human equipotent dosing: rifampin (10 mg/kg/day), isoniazid (10 mg/kg/day), pyrazinamide (25 mg/kg/day), pretomanid (Pa50 = 200 mg/day; Pa100 = 400-600 mg/day; Pa200 = >600 mg/day), bedaquiline (standard oral dosing), linezolid (1,200 mg/day), sutezolid (1,200 mg/day), moxifloxacin (Mx100 = 400 mg/day; Mx200 = ∼800 mg/day), and clofazimine (100 mg/day). Adjunctive dexamethasone (standard of care for TB meningitis for the first 6-8 weeks) was administered to mice via intraperitoneal injection (to match human equipotent dosing) for all regimens. Data are presented as mean ± standard deviation on a logarithmic scale (log10). Each dot represents an individual animal. Animal studies were approved by the Johns Hopkins Animal Care and Use Committee.

**Conclusion:**

Bactericidal activity of antibiotics is substantially different in lung and brain compartments, due discordant tissue exposures. This has important implications for developing antibiotic treatments for TB meningitis. Imaging provides a clinically translatable platform to facilitate antibiotic drug development. These optimized antibiotic regimens should be evaluated in clinical studies for TB meningitis.

**Disclosures:**

**Bhavatharini Arun, PharmD**, Pumas AI Inc.: Employee **Alvaro A. Ordonez, MD**, Fujirebio Diagnostics, Inc, USA: Grant/Research Support **Vijay Ivaturi, PhD**, Pumas AI Inc.: Ownership Interest **Sanjay K. Jain, MD**, Fujirebio Diagnostics, Inc, USA: Grant/Research Support|Novobiotics LLC, USA: Advisor/Consultant|Novobiotics LLC, USA: Grant/Research Support|T3 Pharma, Switzerland: Grant/Research Support

